# Professionals’ and Students’ Perceived Needs for an Online Supportive Application for Reducing School Absence and Stimulating Reintegration: Concept Mapping Study

**DOI:** 10.2196/24659

**Published:** 2021-06-21

**Authors:** Mariette H H Hoogsteder, Linda N Douma, Charlotte G A Eskens, Renske L Berendsen, Yvonne T M Vanneste, Frederieke G Schaafsma

**Affiliations:** 1 Department of Public and Occupational Health Amsterdam Public Health Research Institute Amsterdam University Medical Centers (UMC), Vrije Universiteit Amsterdam Amsterdam Netherlands; 2 GGD (Public Health Service) Flevoland Almere Netherlands; 3 De Zorgcirkel Purmerend Netherlands; 4 Ksyos Telemedical Centre Amstelveen Netherlands; 5 Dutch Knowledge and Innovation Centre Youth and Health (NCJ) Utrecht Netherlands

**Keywords:** medical absenteeism, secondary education, eHealth, mHealth, mobile health, students, schools, health occupations, youth health physicians, concept mapping

## Abstract

**Background:**

To limit students’ medical absenteeism and premature school dropout in the Netherlands, the Medical Advice for Sick-reported Students (MASS) intervention was developed to enhance collaboration between students, parents, school, and health care professionals. MASS reduces medical absenteeism. However, it does not yet optimally support professionals in monitoring students nor automatically stimulating students’ autonomy regarding their situation.

**Objective:**

This study aimed to identify professionals’ and students’ perceived need for an online supportive application to monitor and reduce absenteeism and stimulate student autonomy and school reintegration.

**Methods:**

Concept mapping sessions were held with professionals (n=23) and secondary school students (n=27) in group meetings or online to identify their perspectives and needs. Multidimensional scaling and hierarchical clustering were done with Ariadne 3.0 software. The resulting concept maps were reclustered and interpreted by 4 researchers.

**Results:**

Three heterogeneous groups of professionals generated 17 clusters (135 unique statements), with a mean importance rating ranging from 2.9 to 4.6 on a Likert scale with scores ranging from 1 to 5. Three heterogeneous groups of secondary school students generated 18 clusters (95 unique statements), with a mean importance rating ranging from 3.2 to 4.6. Professionals considered as most important the following: easily accessible contact with students; supporting, motivating, and rewarding students; monitoring absent students; providing information to students and their parents; exchanging information between professionals. Students considered as most important the following: better teacher-student communication and respect; communication between school professionals on the one hand and parents, other professionals, and students on the other hand; guidance in missed learning materials and tests. Students perceived an online format for support as the obvious option.

**Conclusions:**

Both professionals and students were positive about an online application to support students in dealing with medical absenteeism, especially considering the need for better and easily accessible contact between students and professionals. An eHealth or mobile health (mHealth) application addressing these aspects could stimulate student autonomy and have positive effects on medical absenteeism.

## Introduction

School absenteeism is a serious problem in many countries worldwide [1-3], including the Netherlands. Recent numbers show that, for example, in the United States, at least 13% of all students (5-18 years old) are chronically absent from school, involving both excused and unexcused absences as well as suspensions. In the Netherlands, no clear-cut definition of chronic absenteeism exists. Problematic absenteeism is defined as being absent from school for a minimum of 16 hours during a consecutive period of 4 weeks [4]. Recent numbers show that at least 5% of all students aged 5 to 18 years old is problematically absent from school [5,6]. However, this only involves unexcused absences and not absences because of illness or problems. Although Dutch schools are required to register both unexcused and excused absences, they only need to report chronic unexcused absences to the authorities [4]. Frequent and prolonged school absenteeism due to either unexcused or excused absences can have a significant impact on a student’s life, for example leading to stagnation of a student’s (learning) development, a lower education level, and even school dropout [7-10]. In the Netherlands, 2% of students attending secondary or vocational schools (12-23 years old) drop out prematurely, a number that is slowly increasing [5,6,11]. Successfully completing school and having a degree is important, as it will provide better employment opportunities, contributing to a better financial and living situation. Furthermore, people with a school diploma are less likely to exhibit delinquent behavior and more likely to continue studying and to live healthier and longer lives [12-18]. Subsequently, health outcomes can be improved by early diagnosis and management of specific physical and mental health problems associated with school absence [19] and by optimizing educational opportunities [20]. Therefore, school absenteeism and school dropout should be considered public health problems [13,15,16,18].

**Figure 1:**
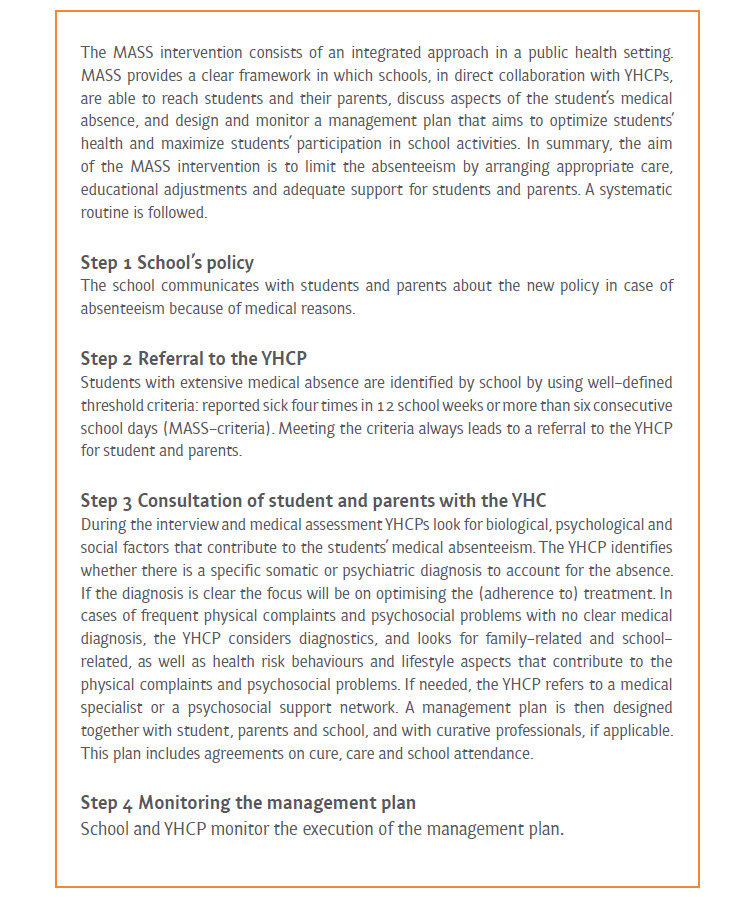
Description of the Dutch intervention “Medical Advice for Sick-reported Students” (MASS) by Vanneste et al [25]. YHCP: youth health care provider.

Behind excused school absenteeism there may be many reasons, medical absenteeism being one of them. Medical absenteeism refers to students reporting absent from school because of (a wide range of) health-related problems [1,12]. Exact numbers relating to medical absenteeism are unclear, as schools are not required to report these to the Dutch authorities. Multiple factors are involved in chronic absenteeism, and schools may differ in how they report absence [4,6,12]. However, a World Health Organization report from 2002 [21] showed that, in half of the school absenteeism cases in Dutch secondary schools, being ill was reported as the cause. Additionally, a national health monitor study with students in the second and fourth grades of secondary school showed that in 40% of absentee cases, students reported that this was because of illness, while truancy was reported in 11% of the cases [22]. This indicates that, in secondary school, medical absenteeism is a significant and prevalent type of absenteeism. However, in most countries, the approach to reducing school absenteeism primarily focuses on truancy. Nonetheless, integrated solutions are being developed such as the involvement of physicians and (public) health services [1,7,12]. In the Netherlands, an integrated approach to addressing medical absenteeism has been developed, namely the Medical Advice for Sick-reported Students (MASS) intervention [23,24] (see Figure 1 by Vanneste et al [25]). This intervention, as well as other school health care activities, is coordinated by the youth health care section of regional Public Health Services. The MASS intervention has dual purposes: (1) stimulating collaboration between professionals involved in the care of a medically absent student, in particular schools, youth health care, and the school attendance service, and (2) optimizing students’ health, maximizing students’ participation in school activities, and limiting school absenteeism for medical reasons [17,18,24,25].

The MASS intervention shows promising effects regarding the reduction of absenteeism in secondary schools for medical reasons [25-27]. However, certain aspects of the intervention could still be further improved, for example, regarding the monitoring of students and stimulating students’ autonomy regarding their situation, as well as regarding the collaboration between professionals [28]. Many different professionals are involved in the MASS intervention and the care and support of students frequently absent from school. This makes collaboration and the alignment of efforts more complex, but also more necessary [29]. eHealth and particularly mobile health (mHealth) could be used to support communication and collaboration between all professionals involved [30,31]. Additionally, it could have beneficial effects for students. According to the European Commission, mHealth has a preventive function and adds value to patient-focused care [32]. mHealth has a broad reach, can be tailored, is easily accessible, appeals to a person’s autonomy, and is relatively low in cost [28,30,33]. Furthermore, mHealth is considered a low threshold tool to access information, change behavior, and monitor and guide a person from a distance [28]. All these aspects would make eHealth or mHealth an appealing tool for both professionals and students to use as support in reducing school absenteeism and stimulating student autonomy, especially in the Netherlands, where over 93% of students use the internet daily [34]. Therefore, a supportive online application might be a helpful tool to complement the MASS intervention and enhance its effects regarding the reduction of school absenteeism and school dropout.

To develop a relevant and effective online application to complement the MASS intervention, it is key to first explore and take into account the perspectives and needs of the target population [35,36]: professionals in the field of school absenteeism and students. Therefore, our study focused on the following research questions:

1. What are the perspectives and needs of professionals involved in the care of an absent student concerning an online application aimed at reducing school absenteeism, stimulating student autonomy, and stimulating school reintegration?
2. What are the perspectives and needs of students concerning an online application aimed at reducing school absenteeism, stimulating student autonomy, and stimulating school reintegration?

## Methods

### Design

This study followed an exploratory descriptive design. To examine the perceptions of relevant stakeholders, the participatory research method of concept mapping was used [37,38]. Concept mapping has been widely applied in health care–related research for this purpose [39]. Concept mapping consists of 6 steps: (1) preparation, (2) generation of statements, (3) structuring of statements, (4) analysis and representation of statements in the form of a concept map, (5) interpretation of the maps, (6) utilization of maps. A qualitative method is used to collect group data (ie, perspectives/statements from the group) following an inductive and structured approach. Quantitative analysis is then used to generate a representation of the collected statements in the form of a concept map. To interpret the generated concept maps, qualitative analysis is used.

### Recruitment of Participants

For our study, we recruited professionals involved in the care or support of absent students, as well as students currently attending secondary school. Concept mapping sessions were conducted with both target groups independently from each other to ensure that they could speak freely, as the hierarchy between students and professionals may affect the group dynamics and responses given. All concept mapping sessions were facilitated by CE and RB. Regarding both target groups, we aimed for a heterogeneous group of participants, to stimulate discussion and provide a more comprehensive and diverse range of perspectives. Participants for both target groups were recruited in the period of February 2018 and March 2018.

With the Public Health Service Flevoland (GGD Flevoland) as our base, we recruited professionals and students from the 2 largest cities in the Flevoland province: Almere (205,000 inhabitants) and Lelystad (77,000 inhabitants) [40].

#### Professionals

The professionals were selected through purposive sampling, aiming to include 30 professionals with diverse job functions from various organizations relevant to (medical) school absenteeism or the MASS intervention (eg, youth health care physicians, teachers, school care coordinators, remedial educationalists, other health professionals, and school attendance officers). All professionals were recruited by email. Follow-up telephone calls were conducted with those who were nonresponsive to the initial email.

#### Students

To recruit students, we first contacted secondary schools—varying in educational levels—located in Almere and Lelystad by email. Follow-up telephone calls were conducted with those that were nonresponsive to the initial email. Eventually, 3 secondary schools were willing to participate. Once a school agreed to participate, the school sent their students a pamphlet and information letter concerning our study. Participants were promised a gift voucher of €10 after completion of the data collection.

### Ethics Approval

The study protocol did not need approval by a Medical Ethics Review Committee because it did not fall within the legal scope of medical research involving human subjects. All students received an informational letter, and written informed consent was obtained before participation. If a student was under the age of 16 years, a parent or caregiver received an informational letter concerning our study as well and had to cosign the informed consent form.

To ensure anonymity and privacy of the participants, personal data and informed consent forms were stored in a secured file only accessible by the researchers. Other data such as fieldnotes and online input per email during the concept mapping were de-identified, removing names and other identifying data if necessary. All data were stored and processed on a secured network drive at the university.

### Concept Mapping Sessions

All sessions followed a similar structure, using the 6 steps involved in concept mapping. We organized 3 concept mapping sessions with professionals (maximum of 2 hours). The first 2 sessions were physical meetings, and the third one was conducted online. We also conducted 3 concept mapping sessions with students at the 3 school locations. However, with Hidding et al [41] as an example, the concept mapping sessions conducted with the students were split into 2 parts because their attention spans can be more limited than that of adults. The first part (1 hour) consisted of the generation of statements (step 2 of concept mapping). The second part (1 hour) consisted of the structuring of statements (step 3). Between the first and second part was an intermission of 1-3 weeks.

#### Step 1: Preparation

Participants were selected, and the focus statement was formulated. A focus statement is used as a starting point in order to stimulate participants to “free associate” and to generate as many perspectives or statements as possible. The focus statement for the professional sessions was: “How can you support young people who are frequently or long-term (medically) absent from school with the help of a digital tool?”

The focus statement for the student sessions was intentionally formulated more broadly in order to explore their overall supportive needs and not only their need for an online application. Additionally, to enhance the understanding of the student participants, the same focus statement was formulated in 2 ways: (1) “How do I want school to help me when I am frequently or long-term ill?” and (2) “When I am frequently or long-term ill, I want school to help me with …”

#### Step 2: Generation of Statements

Each concept mapping session started with an introduction to the topic and concept mapping. Then, participants were asked to respond to the focus statement and to generate as many statements as possible. Participants started with an individual brainstorm of approximately 10 minutes, after which they shared their responses in a group brainstorm. Participants were asked to share 1 statement at a time, after which the other participants could add something to this statement. In the online session (only with professionals), all the statements of the individual brainstorm were sent by email to the facilitators, who merged all the statements and subsequently shared all the statements with the group. Then, via email, participants could respond to the given statements and add new ones. Regarding all sessions, the final list of generated statements was reformulated, where necessary, by the session facilitators (CE and RB and discussed with MH and FS until consensus was reached) to keep the meaning of the statements clear and understandable for every participant.

#### Step 3: Structuring of Statements

The structuring of statements was conducted using the concept mapping software Ariadne 3.0 [42]. Participants first individually organized all statements generated in their session according to content, grouping statements into clusters of related statements. Subsequently, they each ranked the importance of all statements according to a 5-point Likert scale, where 1 represented “not important at all” and 5 represented “very important.”

#### Step 4: Representation of Statements in the Form of a Concept Map

Using Ariadne 3.0, a graphical representation of the statements was created in the form of a concept map [39]. All statements are presented as separate points in the visual representation/concept map. Statements that are frequently placed together are similar in content and presented more closely together on the concept map, resulting in clusters. Statements that are seldomly placed together by the participants are placed on the map opposite from each other. The analyses were conducted separately for each concept mapping session.

#### Step 5: Interpretation of the Maps

The interpretation of the final concept maps, in which the clusters (ie, themes) are named and discussed, was done by 4 researchers (CE, RB, FS, and MH). Similarities between statements in every cluster were identified. The researchers named each cluster in the final concept maps by the overarching theme that it represented. Subsequently, the researchers critically contemplated the clusters generated by the software Ariadne 3.0, because some of the statements did not optimally suit a particular cluster. After deliberation and when consensus between the researchers was reached, some of the statements were moved to other clusters nearby, or new clusters were formed. Finally, using Ariadne 3.0, the average importance rates of the clusters were calculated to rank them from high to low.

#### Step 6: Utilization of Maps

The sixth and final concept mapping step, utilization of the maps, involves the identification of determinants that could be used for the development of a supportive mobile online application to complement the MASS intervention (see Discussion).

### Statistical Analysis

The data from each concept mapping session were analyzed with the software program Ariadne 3.0 [42], which uses multivariate statistical techniques (multidimensional scaling and hierarchical clustering) to visually present statements generated in the group sessions. The analyses resulted in 1 concept map per session.

## Results

### Participants

#### Professionals

Overall, 23 professionals participated in our concept mapping sessions (Table 1). For the first session, 28 professionals were invited, of which 5 were able to participate. For the second session, 17 professionals were invited, of which 10 were able to participate. The third (online) session consisted of 8 professionals who were initially invited for the first or second session but could not participate earlier because of a busy work schedule. In this session, only 6 participants were involved with the clustering and prioritizing of the statements (step three of concept mapping), again because of a busy work schedule. The majority of our sample was female (n=21), ranging from 27 to 60 years old and with an average work experience with school absenteeism of 9 (SD 6.4) years. The participants were professionals with diverse functions in 7 organizations and 6 schools (see Multimedia Appendix 1 for an overview of the professionals’ job functions).

**Table 1:** Sample characteristics of professionals (n=23).

Characteristic			n (%)
**Gender**			
	Male	2 (9)	
	Female	21 (91)	
**Age group (years)**			
	21-30	4 (17)	
	31-40	6 (26)	
	41-50	8 (35)	
	51-60	5 (22)	
**Work experience with school absenteeism (years)**			
	<5	8 (35)	
	5-10	6 (26)	
	11-20	8 (35)	
	>20	1 (4)	

#### Students

Overall, 27 students participated in our study (Table 2), ranging in age from 13 to 18 years old, with the majority being female (n=16). They attended 1 of the 3 participating secondary schools: (1) a special education school teaching at the general secondary level (n=7); (2) a regular school teaching at various educational levels (n=11); (3) a regular school teaching at the prevocational secondary level (n=9). The MASS criteria were met by 8 participants (see Figure 1, Step 2 [24,25]) of school absenteeism for medical reasons. Although 4 participants, of which 3 met the MASS criteria, were not able to attend the second part of the concept mapping session, 1 of them completed the session online.

**Table 2:** Sample characteristics of students (n=27).

Characteristic			n (%)
**Gender**			
	Male	11 (40)	
	Female	16 (60)	
**Age (years)**			
	13	5 (18)	
	14	3 (11)	
	15	1 (4)	
	16	10 (37)	
	17	7 (26)	
	18	1 (4)	
**Educational level**			
	Pre-vocational secondary (Dutch: vmbo)	16 (59)	
	Higher general secondary (Dutch: havo)	7 (26)	
	Pre-university (Dutch: vwo)	3 (11)	
	Unknown	1 (4)	
Special education (yes)			7 (26)
Meeting MASS^a^ criteria (yes)			8 (30)

^a^MASS: Medical Advice for Sick-reported Students.

### Concept Maps and Clusters

#### Professionals

Three heterogeneous groups of professionals generated 135 unique statements, with an importance rating ranging from 1.6 to 4.8 on the Likert scale with scores ranging from 1 to 5. These statements were combined into 17 clusters, with a mean importance rating ranging from 2.9 to 4.6 on the Likert scale with scores ranging from 1 to 5 (see Multimedia Appendix 2 for the corresponding concept maps). Table 3 presents the clusters relating to the perspectives and needs for a supportive tool in reducing school absenteeism mentioned by professionals. Important aspects as considered by professionals were (1) easily accessible contact with students; (2) supporting, motivating, and rewarding students; (3) monitoring absent students; (4) providing information to students and their parents; and (5) exchanging information between professionals.

**Table 3:** Clusters, associated example statements, and mean importance ratings per group concerning the perspectives and needs of professionals for a supportive tool in reducing school absenteeism.

Cluster name			Example statement		Mean importance^a^
**Group 1**					
	1. Contact from (school) professional to student	An extra function for mentors to get in contact with the students		4.6	
	2. Reward system	A reward system when showing desired behavior		4.2	
	3. Contact from student to (school) professional	The possibility for students to have easily accessible contact with the outside world		3.8	
	4. Characteristics and functions of the application	User-friendly for all school levels		3.7	
	5. Exchange information between professionals	An overview of concerned parties to improve the collaboration between professionals		3.5	
	6. Responsibilities of involved youth health care professionals	Help from the youth health care nurse during start-up for students to use the application		3.2	
	7. Inform students and parents about school absenteeism	To provide information about the consequences of school absenteeism, including appropriate tools		3.2	
**Group 2**					
	1. Dossier access for professionals	Access to the student’s dossier so all involved parties can add supplemental information		4.1	
	2. Contact with students and other features of the application	Direct lines with the students to make them feel noticed		3.7	
	3. Monitor absent students	A system to map the (absenteeism) developments of the students		3.7	
	4. A separate account for parents	A notification for parents about their child’s school absenteeism		3.2	
	5. Provide information and an overview of professionals involved	An overview for students to understand precisely what is and is not a legitimate reason for school absenteeism		2.9	
**Group 3**					
	1. Contact between (school) professionals and students	Daily or more frequent contact with students who are absent		4.3	
	2. Support and motivate students	Inclusion of self-set goals by the students in the tool, with indicators if they are achieved or not		3.7	
	3. Responsibilities of schools and other professionals	Prevent students from long-term absenteeism by supporting them in formulating their action plan and goals		3.5	
	4. Provide information and monitor absenteeism	To provide students with advice, tailored to their problems, such as problems with eating, sleeping, gaming, or mood swings		3.5	
	5. Involve parents and professionals	Support and unburden parents using the tool		3.1	

^a^Rated on a 5-point Likert scale with higher scores indicating higher importance.

The first aspect, easily accessible contact with students, refers to the professionals’ reported need for a supportive online tool to be able to communicate and stay in contact with absent students in an easy and low-threshold way. Professionals expressed the belief that students should receive attention and support in an approachable and informal manner. Additionally, the application design should be appealing for and tailored to the target group of students.

The second, third, and fourth aspects (supporting, motivating, and rewarding students; monitoring absent students; and providing information to students and their parents, respectively) were closely related to each other, with all emphasizing the importance of adequately supporting, motivating, and monitoring students. Professionals expressed the importance of working with personal goals and a reward system when students show the behavior that brings them closer to their personal goals, for example, when they are present at school or doing schoolwork at home. Additionally, suggestions were made to add a game or reward element to motivate students to work on their goals and to continue using the tool. The ability to monitor students using a supportive tool would provide professionals with more insight into the frequency of school absenteeism and to what extent absent students experience physical and psychological symptoms. In addition, professionals mentioned a preference for a separate account for parents, enabling them to support their child with working on their goals, after their child gave permission. Furthermore, professionals expressed a need to provide information to both students and parents through an online tool, especially about the causes and consequences of school absenteeism and how to deal with common health and learning issues in general.

The fifth aspect, exchanging information between professionals, refers to professionals wanting to know which other care organizations or professionals are involved with a student and to be able to share relevant information with them—in a secure manner and with the student’s informed consent—in order to improve collaboration and support for the student. The suggestion was to use the online tool to provide an overview and contact details of the professionals involved.

#### Students

Three heterogeneous groups of secondary school students generated 95 unique statements, with an importance rating ranging from 2.2 to 4.9 on the Likert scale with scores ranging from 1 to 5. These statements were combined into 18 clusters, with a mean importance rating ranging from 3.2 to 4.6 on the Likert scale with scores ranging from 1 to 5 (see Multimedia Appendix 3 for the corresponding concept maps). Table 4 presents the clusters relating to the perspectives and needs for a supportive tool in reducing school absenteeism mentioned by students. Important aspects as considered by students were (1) respect and better teacher-student communication; (2) communication between school on the one hand and parents, professionals, and students on the other hand; and (3) guidance for missed learning materials and tests. They perceived an online format as a supportive tool as undoubtedly the obvious option.

**Table 4:** Clusters, associated example statements, and mean importance ratings per group concerning the perspectives and needs of students for a supportive tool in reducing school absenteeism.

Cluster name		Example statement	Mean importance^a^
**Group 1**			
	1. Training teachers	Training teachers to support students in returning to school	4.2
	2. Counsellor	You should get along with your counsellor	3.8
	3. Information for parents	Overview for parents of when their child has to go and does not have to go to school	3.6
	4. Dossier	Information in the dossier is confidential and well-protected	3.6
	5. Missed learning materials and tests	Review of missed learning materials provided by school	3.4
	6. Contact between teachers and students	Teachers are better informed on what is going on with the student	3.2
	7. Independent case management	Improve and expedite the communication between school and attendance officer	3.2
**Group 2**			
	1. Communication and respect	Equal rights and rules for students and teachers	4.6
	2. Registration of medical absence	Easier logging in and out	4.4
	3. Schedule	Access to correct school schedule	4.4
	4. Overview of missed learning materials	Online summary of missed learning materials available	4.2
	5. Alleviating the rules on absenteeism	Maintaining the opportunity to do a makeup test when you missed a test due to illness	4.1
	6. Preparations for a test	Having the option to review a test before the next one to be able to prepare better	3.9
	7. Communication during absenteeism	Weekly check-in by school how the absent student is doing	3.6
**Group 3**			
	1. Keeping up with learning materials and more fun classes	More fun classes help to pay attention and to return to school	4.0
	2. Respect and rules on absenteeism	Teachers have to treat students respectfully	3.8
	3. Online features	One working app that displays the schedule, homework, grades, and missed information	3.6
	4. Makeup tests	Offering makeup tests for a longer period of time	3.2

^a^Rated on a 5-point Likert scale with higher scores indicating higher importance.

The first 2 aspects (respect and better teacher-student communication; communication between school on the one hand and parents, professionals, and students on the other hand) refer to students’ reported need for teachers to be better informed about a student’s situation and well-being and to improve communication between all parties involved (ie, school, students, parents, other professionals). Students expressed a desire for equal rights, rules, and respect between teachers and themselves and a preference to be able to ask their teacher questions through an easily accessible (online) tool. Students also believed that schools should ask in-depth questions about their reason for their absence and expressed a need for earlier communication by the school regarding a possible change in their schedule.

The third aspect (guidance for missed learning materials and tests) refers to students’ expressed need for an overview of the missed learning materials and a main contact person from which to receive relevant notes, learning materials, and homework. Additionally, students expressed a desire for take-home tests and an extension of the period to take tests that they missed. The fourth aspect (online support as obvious) refers to the students’ preferred supporting format, namely online. Students mentioned that one comprehensive application is necessary, providing them access to their schedule, homework, grades, and (missed) learning materials. They also discussed the need for a forum where they can post questions to their peers and teachers.

## Discussion

### Principal Findings

Both professionals and students expressed positive perceptions and identified added value regarding an online application to support students in dealing with school absenteeism for medical reasons and to stimulate student autonomy and return to school. The most important aspects of a supportive eHealth or mHealth application as considered by professionals were easily accessible contact with students; supporting, motivating, and rewarding students; monitoring absent students; providing information to students and their parents; and exchanging information between professionals. The most important aspects as considered by students were better teacher-student communication and respect; communication between school on the one hand and parents, professionals, and students on the other hand; and guidance for missed learning materials and tests. Furthermore, students perceived an online format as a supportive tool as undoubtedly the obvious and self-evident option.

A shared need among professionals and students in our study was the fostering of better and easily accessible contact between students, parents, and professionals involved, preferably using online facilities. Previous research on the needs of health care professionals relating to patient care and the use of eHealth or mHealth also found easily accessible contact to be an important need [35,43]. Additionally, studies reviewing the impact of mHealth interventions worldwide reported that the use of mHealth can improve communication with and between health care professionals [30,44]. Furthermore, previous research within the context of school absenteeism and dropout also confirms the importance of easily accessible contact between students, parents, and professionals. Positive teacher-student contact contributes to students feeling supported, empowered, and engaged in school, as well as a reduction of school absenteeism [45-48]. e-Learning and other online tools can facilitate establishing this contact and related benefits [49,50]. In addition, better communication between schools and parents contributes to parental involvement, which has shown to be beneficial to students’ school engagement and reduced absenteeism [46,51,52]. The benefits of better and easily accessible contact between all parties involved relate to the monitoring, supporting, motivating, and guiding of students in general, which are all aspects of a supportive online application considered important by professionals and or students in our study.

The importance of easily accessible contact is also supported by research on occupational health [53-55], which appears to have relevant similarities to the context of school absenteeism. From the occupational health research, it is known that keeping in touch with medically absent workers on a regular basis and providing supervisor or co-worker support helps with the return to work or being able to continue working when chronically ill [53]. Additionally, these studies have shown that it is beneficial for the absent worker to discuss returning to work with their employer and to receive information on the recovery process as well as on sick leave procedures. Also, if necessary, an occupational health physician will be involved [54,55]. For an absent worker, it is just as essential as it is for students that contact and communication between all parties involved is done with respect, taking the perspective of the absentee as a starting point [53,55].

The perceptions and needs expressed by professionals and students in our study relate to key elements of the MASS intervention, particularly communication, motivation, individual monitoring/guidance, early detection of absence, and professional collaboration [24-27], with the addition of incorporating online facilities. The MASS intervention is promising in reducing medical absenteeism in secondary schools [25-27], but aspects such as the monitoring of students, stimulating students’ autonomy, and collaboration between professionals could be further improved [28]. The findings from our study indicate that these specific aspects could be addressed with the use of an online application, which is supported by many previous studies showing eHealth or mHealth and online tools to have an empowering and facilitating effect [28,30-33,50,56-59]. Therefore, developing an online application complementary to the MASS intervention could potentially stimulate and enhance the intervention’s positive effects on school absenteeism, especially when the online application can be tailored to students’ unique sets of reasons behind their absenteeism and their individual needs [45].

### Strengths and Limitations

To our knowledge, this is the first study to apply concept mapping to examine professionals’ and students’ perspectives regarding (medical) school absenteeism as well as the use of an online tool. Our study has several strengths. First, our study samples of professionals and students were relatively heterogeneous (differing in age, education level, experience with school absenteeism, school location, organization, job function, and work experience), increasing the generalizability of our findings. Second, combining individual generation of ideas with a group discussion of these ideas, as well as the context of heterogeneous groups, results in an ample and diverse selection of ideas, thoughts, and perceptions. Third, the concept mapping method has the added value of structuring qualitative data in a quantitative manner and visualizing the results.

Our study also has some limitations. First, generalizability could be affected because we only included participants from 1 of 12 Dutch provinces and most student participants were not randomly selected by their school. Also, professionals with—or experiencing—a heavier workload may have been underrepresented, as a busy work schedule was the most common reason not to participate. Additionally, the sample sizes of 2 of the professional concept mapping sessions were smaller than the recommended minimum number of 8 participants [42]. However, other studies using smaller sample sizes have been successful in applying the concept mapping method [60,61]. Furthermore, the online format used with the third group of professionals resulted in less interaction between professionals compared to the physical group meetings, potentially restricting the generation of new ideas. Nevertheless, this online concept mapping session generated the most statements of all sessions with professionals. It is possible data saturation regarding the professionals’ perspectives has not been reached, although the themes generated in both the online and physical sessions were similar to each other. Due to time restrictions as well as the busy schedules of professionals, an additional concept mapping session was not feasible within our current study. Data saturation regarding the students’ perspectives does appear to have been reached, as only one new theme emerged during the third concept mapping session with students.

### Future Activities

The sixth and final concept mapping step, utilization of the maps, was not conducted in this study. However, the development of an actual online application, in which the perspectives of both professionals and students will be included, was already planned. The interpretation of clusters and concept maps generated in our current study proved helpful in deciding which statements or themes to include in the development of the online application. A selection of these themes will be translated into functionalities for a pilot version of the application that will be tested and evaluated with professionals and students from more cities and provinces than in our current study. We consider the online application development a co-creation process, collaborating with relevant stakeholders from needs assessment to development, testing, and (effect) evaluation [62,63].

### Conclusion

Both professionals and students were positive about an online application to support students in dealing with school absenteeism for medical reasons and to stimulate student autonomy and return to school. Better and easily accessible contact between students, parents, and professionals was a shared need for which an online tool is highly suited. Developing an online application to be used complementary to the MASS intervention could potentially stimulate and enhance the intervention’s positive effects on school absenteeism.

## Appendix

Multimedia Appendix 1 Overview of professionals’ job functions (total N=23).

Multimedia Appendix 2 Concept maps of the sessions with professionals.

Multimedia Appendix 3 Concept maps of the sessions with students.

## Abbreviations

MASS: Medical Advice for Sick-reported Students
mHealth: mobile health
