# Supplementary material for: Professionals’ and Students’ Perceived Needs for an Online Supportive Application for Reducing School Absence and Stimulating Reintegration: Concept Mapping Study
Source: JMIR Form Res. 2021 Jun 21;5(6):e24659. doi: 10.2196/24659 (PMC8277345; doi:10.2196/24659)
Supplement: Multimedia Appendix 1 [file formative_v5i6e24659_app1.docx]

**Appendix 1. Overview of professionals’ job functions (total *N* = 23)**

| **Job function** | **N (%)^*^** |
| --- | --- |
| School care coordinator | 6 (26) |
| Youth health care physician | 4 (17) |
| School attendance officer | 3 (13) |
| Policy officer¹ | 3 (13) |
| Remedial educationalist | 2 (9) |
| Youth behavioural expert | 1 (4) |
| Youth health care nurse | 1 (4) |
| Social educational aid worker | 1 (4) |
| Team leader² | 1 (4) |
| Department leader³ | 1 (4) |

¹ Working in the area of youth care or premature school drop-out

² Working at an organization that supports schools

³ Working at a special education secondary school

* Total amount is less than 100% due to rounding
