# Supplementary material for: Professionals’ and Students’ Perceived Needs for an Online Supportive Application for Reducing School Absence and Stimulating Reintegration: Concept Mapping Study
Source: JMIR Form Res. 2021 Jun 21;5(6):e24659. doi: 10.2196/24659 (PMC8277345; doi:10.2196/24659)
Supplement: Multimedia Appendix 2 [file formative_v5i6e24659_app2.docx]

**Appendix 2. Concept maps of the sessions with professionals**

NB. As a standard, Ariadne 3.0 generates eight clusters, irrespective of the actual number of clusters generated by the participants. However, it is possible, if there is sufficient reason to do so, to manually generate more or less clusters by merging or separating them. After deliberation and when consensus between the researchers was reached, some of the statements were moved to other clusters nearby or new clusters were formed.

**Figure B1.** Concept map of the first session with professionals. Note: the points show the statements, an arrow a reallocated statement and a circle a newly formed cluster by researchers. Cluster 1: Contact from (school) professional to student; Cluster 2: Reward system; Cluster 3: Contact from student to (school) professional; Cluster 4: Characteristics and functions of the application; Cluster 5: Exchange information between professionals; Cluster 6: Responsibilities of involved youth health care professionals; Cluster 7: Inform students and parents about school absenteeism.

**Figure B2.** Concept map of the second session with professionals. Note: the points show the statements, an arrow a reallocated statement and a circle a newly formed cluster by researchers. Cluster 1: Dossier access for professionals; Cluster 2: Contact with students and other features of the application; Cluster 3: Monitor absent students; Cluster 4: A separate account for parents; Cluster 5: Provide information and an overview of professionals involved.

**Figure B3.** Concept map of the third session with professionals (online). Note: the points show the statements, an arrow a reallocated statement and a circle a newly formed cluster by researchers. Cluster 1: Contact between (school) professionals and students; Cluster 2: Support and motivate students; Cluster 3: Responsibilities of schools and other professionals; Cluster 4: Provide information and monitor absenteeism; Cluster 5: Involve parents and professionals.
