# Supplementary material for: Professionals’ and Students’ Perceived Needs for an Online Supportive Application for Reducing School Absence and Stimulating Reintegration: Concept Mapping Study
Source: JMIR Form Res. 2021 Jun 21;5(6):e24659. doi: 10.2196/24659 (PMC8277345; doi:10.2196/24659)
Supplement: Multimedia Appendix 3 [file formative_v5i6e24659_app3.docx]

**Appendix 3. Concept maps of the sessions with students**

NB. As a standard, Ariadne 3.0 generates eight clusters, irrespective of the actual number of clusters generated by the participants. However, it is possible, if there is sufficient reason to do so, to manually generate more or less clusters by merging or separating them. After deliberation and when consensus between the researchers was reached, some of the statements were moved to other clusters nearby or new clusters were formed.

**Figure C1.** Concept map of the first session with students. Note: the points show the statements, an arrow a reallocated statement and a circle a newly formed cluster by researchers. Cluster 1: Training teachers; Cluster 2: Counsellor; Cluster 3: Information for parents; Cluster 4: Dossier; Cluster 5: Missed learning materials and tests; Cluster 6: Contact between teachers and students; Cluster 7: Independent case management.

**Figure C2.** Concept map of the second session with students. Note: the points show the statements, an arrow a reallocated statement and a circle a newly formed cluster by researchers. Cluster 1: Communication and respect; Cluster 2: Registration medical absence; Cluster 3: Schedule; Cluster 4: Overview missed learning materials; Cluster 5: Alleviating the rules on absenteeism; Cluster 6: Preparations for a test; Cluster 7: Communication during absenteeism.

**Figure C3.** Concept map of the third session with students. Note: the points show the statements, an arrow a reallocated statement and a circle a newly formed cluster by researchers. Cluster 1: Keeping up with learning materials, and more fun classes; Cluster 2: Respect and rules on absenteeism; Cluster 3: Online features; Cluster 4: Makeup tests.
